# Prostate-Specific Membrane Antigen (PSMA) PET/CT in the Detection and Diagnosis of Hepatocellular Carcinoma (HCC): A Systematic Review and Meta-Analysis

**DOI:** 10.3390/cancers16223865

**Published:** 2024-11-19

**Authors:** Nicholas Hannah, Catherine Yu, Leya Nedumannil, James Haridy, Grace Kong, Alex Boussioutas, Siddharth Sood

**Affiliations:** 1 Melbourne Medical School, The University of Melbourne, Parkville, Melbourne, VIC 3052, Australiasiddharth.sood1@nh.org.au (S.S.); 2 Department of Gastroenterology & Hepatology, The Royal Melbourne Hospital, Melbourne Health, Parkville, Melbourne, VIC 3052, Australia; 3 Department of Gastroenterology & Hepatology, The Northern Hospital, Northern Health, Epping, Melbourne, VIC 3076, Australia; 4 Molecular Imaging and Therapeutic Nuclear Medicine, Cancer Imaging, Peter MacCallum Cancer Centre, Melbourne, VIC 3002, Australia; 5 Sir Peter MacCallum Department of Oncology, University of Melbourne, Melbourne, VIC 3002, Australia; 6 School of Translational Medicine, Monash University, 99 Commercial Rd, Melbourne, VIC 3004, Australia; 7 Department of Gastroenterology, The Alfred Hospital, 55 Commercial Rd, Melbourne, VIC 3004, Australia

**Keywords:** hepatocellular carcinoma, HCC, PSMA, PET, diagnosis

## Abstract

Prostate-specific membrane antigen (PSMA) positron emission tomography (PET) is widely used in prostate cancer. Recent studies indicate hepatocellular carcinoma (HCC) demonstrates PSMA PET uptake. The diagnostic accuracy of PSMA PET for HCC is not known. We conducted a systematic review and meta-analysis of studies assessing ^68^Ga-PSMA-11 in HCC. Nine studies were included, with 196 patients and a total of 491 HCC lesions. Per-patient analysis yielded a pooled sensitivity of 89.8% (95% CI 78.5–95.5). Specificity was poorly reported, with insufficient data. When per-lesion level analysis was performed on seven studies, the pooled sensitivity was 94.5% (95% CI 82.9–98.4), and specificity was again poorly reported with insufficient data. Among the three studies with adequate data for full per-lesion meta-analysis, 115 lesions in 41 patients demonstrated sensitivity of 97.1% (95% CI 87.8–99.4), while specificity was 42.2% (95% CI 0.3–99.4). Two studies provided sufficient data for meta-analysis on a per-patient level (*n* = 50 patients), demonstrating a sensitivity of 92.5% (95% CI 64.0–98.9) and specificity of 72.4% (95% CI 1.3–99.8). PSMA PET demonstrates a high sensitivity for HCC and shows promise as an imaging modality for diagnosis and staging of HCC. However, the existing literature does not provide enough data to confidently evaluate its specificity and, therefore, accuracy. Further prospective studies are necessary, with a focus on the accurate reporting of benign lesions and inclusion of patients with an intermediate probability of HCC.

## Simple Summary

Hepatocellular carcinoma (HCC) is a leading cause of cancer-related death world-wide. Prostate-specific membrane antigen (PSMA) positron emission tomography (PET) is a novel imaging modality used in prostate cancer that appears to demonstrate uptake in HCC. The aim of this systematic review and meta-analysis was to determine the sensitivity and specificity of PSMA PET for HCC. On systematic review, ^68^Ga-PSMA-11 demonstrated a high sensitivity on per-patient analysis for HCC, 89.9% (95% CI 78.5–95.5), and on a per-lesion analysis, this increased to 94.5% (95% CI 82.9–98.4). Specificity, however, was poorly reported, with insufficient data for analysis. The meta-analysis of studies with sufficient data demonstrated a sensitivity of 97.1% (95% CI 87.8–99.4) and specificity of 42.2% (95% CI 0.3–99.4) on per-lesion level for HCC. Specificity has been poorly reported, and diagnostic accuracy remains unclear. Further prospective studies are required to define the true specificity of PSMA PET for HCC.

## 1. Introduction

Hepatocellular carcinoma (HCC) is the 6th commonest cancer and the 3rd leading cause for cancer death worldwide [1]. The incidence of HCC and associated mortality is increasing. Early diagnosis of HCC allows for potentially curative therapy, but advanced disease and particularly extra-hepatic spread have a poor prognosis [2].

In established cirrhosis, diagnosis of HCC can be made using radiological imaging with computed tomography (CT) and/or magnetic resonance imaging (MRI). The presence of typical non-rim arterial phase hyperenhancement, venous washout, presence of a capsule, and threshold growth allows for the accurate diagnosis of HCC without the need for histopathological confirmation [2]. Not all liver lesions represent HCC, and the Liver Imaging Reporting and Data System (LI-RADS) was developed to help more comprehensively classify lesions [3]. Lesions that exhibit all the above characteristics are classified as LI-RADS 5, indicating a definitive diagnosis of HCC. Those that lack one or more features are categorized as follows: LI-RADS 4 for probably HCC, LI-RADS 3 for intermediate probability, LI-RADS 2 for probably benign, and LI-RADS 1 for benign.

Prognosis, treatment, and suitability for transplantation depend markedly on features of the tumour such as size, invasion of surrounding structures and extra-hepatic spread. Appropriate staging is essential in providing the best care, and treatment strategy. To date, staging has relied on initial liver imaging along with nuclear medicine bone scans and chest CT. Molecular imaging with Positron Emitting Tomography (PET), using the most common tracer, 2-[18F]fluoro-2-deoxy-D-glucose (^18^F-FDG), has not been widely adopted in the management of patients with HCC. A meta-analysis of retrospective studies reported a pooled sensitivity of 76.6% and specificity of 98% for ^18^F-FDG PET [4]; however, many prospective studies have reported much lower sensitivity <55% [5,6,7]. The poor sensitivity in HCC is likely due to the unique properties of the liver and tumour. High rates of FDG-6-phosphatase activity, high expression of P-glycoprotein, and low GLUT1 and GLUT2 expression in moderately and well-differentiated HCC [8] result in low uptake of FDG into the HCC cell, rapid phosphorylation of FDG, and then efflux of FDG back out of the cell. With limited utility of ^18^F-FDG, a number of other radiotracers have been explored for use in HCC.

Prostate-specific membrane antigen (PSMA) is a transmembrane glycoprotein. Radio-labelled PSMA is increasingly used in the imaging and staging of prostate cancer using PET/CT given the high sensitivity and specificity for PSMA on prostate cancer cells [9,10]. This has been further developed, with the TheraP [11] and VISION [12] trials demonstrating the utility of ^177^Lu-PSMA as a theranostic treatment modality, and due to its improved progression-free and overall survival, it is now utilised in the treatment of metastatic castrate-resistant prostate cancer.

In addition to prostate cancer, there is emerging evidence of high PSMA uptake in other solid tumours [13], including HCC [14,15]. This new imaging modality may aid in more accurate staging of HCC and diagnosis of indeterminate lesions based on current radiological criteria. The aim of this systematic review and meta-analysis is to determine the sensitivity, specificity, and diagnostic accuracy of PSMA PET for HCC.

## 2. Materials and Methods

**Figure 1:**
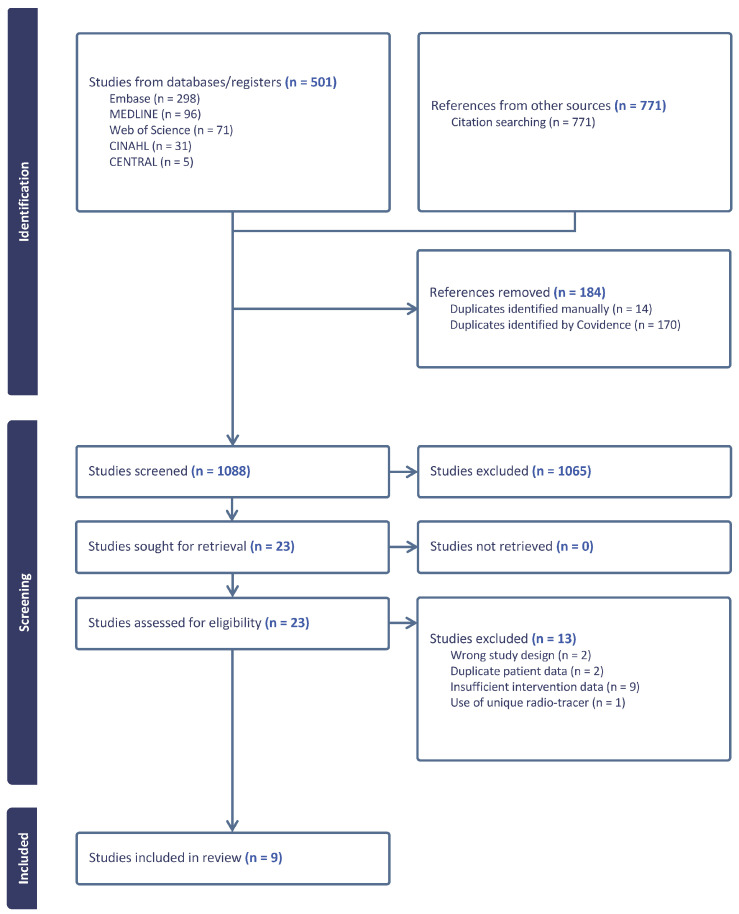
Preferred Reporting Items for Systematic Reviews and Meta-Analysis (PRISMA) flow-diagram.

We performed a systematic review and meta-analysis of the literature as of November 2023. The literature search and resulting records were managed as recorded in the Preferred Reporting Items for Systematic Reviews and Meta-Analysis (PRISMA) diagram (Figure 1). This systematic review was prospectively registered on the NIHR PROSPERO database (Registration number: CRD42023489512).

Searches of biomedical databases and trial registries were designed and run by a clinical librarian. Keywords, synonyms, and controlled vocabulary terms were used. The search strategy was consistent across resources, except for translation of syntax and controlled vocabulary where appropriate (e.g., MeSH terms for Medline were translated to EMTREE terms for EMBASE). Medline (1946 to 2023) and EMBASE (1974 to 2023) were searched via OVID. CINAHL (1937 to 2023) was searched via EBSCO. Cochrane CENTRAL (inception to 2023) was searched via Wiley. Web of Science Core Collection was searched via Web of Science (1900–2023). No language, date, or article-type limits were applied. No filters were used in Cochrane CENTRAL. Searches were run on 30 November 2023. (Full search strategies are available as Supplementary Material). The search strategy’s basic logic is: (LIVER CANCERS AND ((PSMA AND PETCT) OR PSMA PET-CT)). Results of searches were exported as .ris files and imported into Covidence (Covidence systematic review software, Veritas Health Innovation, Melbourne, Australia. Available at www.covidence.org) for deduplication and screening accessed on 7 December 2023. Two reviewers independently screened titles and abstracts of identified studies. Full texts were retrieved and then reviewed for inclusion. Where there was disagreement, a third reviewer reviewed, with subsequent discussion and consensus determining inclusion.

The target population for inclusion consisted of patients with liver lesions in a high-risk demographic, defined by the presence of cirrhosis, hepatitis B virus infection, or a prior history of HCC. The index test of interest was PSMA PET/CT. Standard of truth for a true-positive diagnosis of HCC was a histological diagnosis or Liver Imaging Data Reporting System (LIRADS) 5 lesion. Outcomes of interest in this review were diagnostic sensitivity, specificity, and diagnostic accuracy of PSMA PET for HCC. The types of studies included were randomised controlled trials, prospective and retrospective cohort studies, observational studies, and case series. Case reports and studies including multiple cancers without histological confirmation were excluded, as were studies of PSMA PET in other malignancies.

Data extraction was performed by two independent reviewers in Covidence using the following pre-defined variables: study details, author details, study design, study aim, blinding, time perspective, inclusion criteria, exclusion criteria, group differences, number of patients, baseline clinicopathologic information of patients, baseline imaging CT or MRI, number of lesions on baseline imaging, index test used, number of lesions with uptake on index test, specifications of PSMA tracer used, device, manufacturer, uptake time, administered activity, and study outcomes on per-lesion and per-patient basis: SUVmax tumour, SUVmean tumour, tumour/liver ratio (TLR), true positive, true negative, false positive, false negative, reported sensitivity, reported specificity, reported positive predictive value, and reported negative predictive value.

As a systematic analysis of a diagnostic study, the Quality Assessment of Diagnostic Accuracy Studies QUADAS-2 tool was utilised [16]. Two independent reviewers assessed the risk of bias and applicability of each of the included studies. In cases of disagreement, we reached consensus through discussion.

The sensitivity of radiolabelled PSMA PET was calculated on a per-patient and a per-lesion basis for analysis. The sensitivity was defined as the ratio between the number of HCC patients/lesions detected at radiolabelled PSMA PET and the overall HCC patients/lesions according to the reference standard. Studies included in the statistical pooling investigated the use of PSMA PET/CT for detecting HCC with a diagnosis established by a gold standard test, as outlined above. For a diagnostic test, sensitivity and specificity were considered related to each other. Independent sensitivities and specificities were therefore combined and the SROC created to bypass this problem. Summary estimates for sensitivity (SN), specificity (SP), likelihood ratios (+LR, −LR), and diagnostic odds ratios (DOR) were generated. Quality assessment graphs and forest plots were created utilising free software Review Manager Version: 8.10.0 [17], and analyses were performed and SROC curves generated using MetaDTA (version 2.1.1) [18,19].

## 3. Results

### 3.1. Study Selection

A total of 501 studies were identified in the primary literature search of Embase, MEDLINE, Web of Science, CINAHL, and CENTRAL, and a further 771 references were found via citation searching of the primary studies, giving a total of 1272 studies. Following removal of duplicate entries (*n* = 184), 1088 studies proceeded to title and abstract review. Studies were screened to meet the target population inclusion criteria of either a histological or radiological diagnosis of HCC and patients who underwent PSMA PET/CT or PSMA PET/MRI. Of the 1272 studies, 1065 were excluded, generally due to not meeting the inclusion of HCC. Studies of PSMA PET on other malignancies were also excluded in this review, leaving 23 studies for retrieval and full-text review (Figure 1). Thirteen studies were excluded due to wrong study design (*n* = 2), duplicate patient data (*n* = 2), or insufficient intervention data (*n* = 9). One further study was excluded to reduce the heterogeneity in included radiotracers, as it utilised ^18^F-PSMA-1007 [20], leaving nine studies that utilised the radiotracer ^68^Ga-PSMA-11, which were included in the final analysis [21,22,23,24,25,26,27,28,29]. The first identified clinical study of PSMA PET for detection of HCC was in 2019 in Turkey.

### 3.2. Quality Assessment of Included Studies

**Figure 2:**
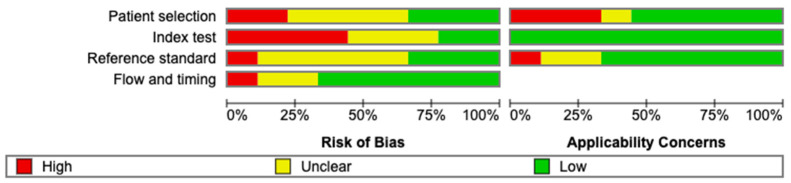
Methodological quality—QUADAS-2.

**Figure 3:**
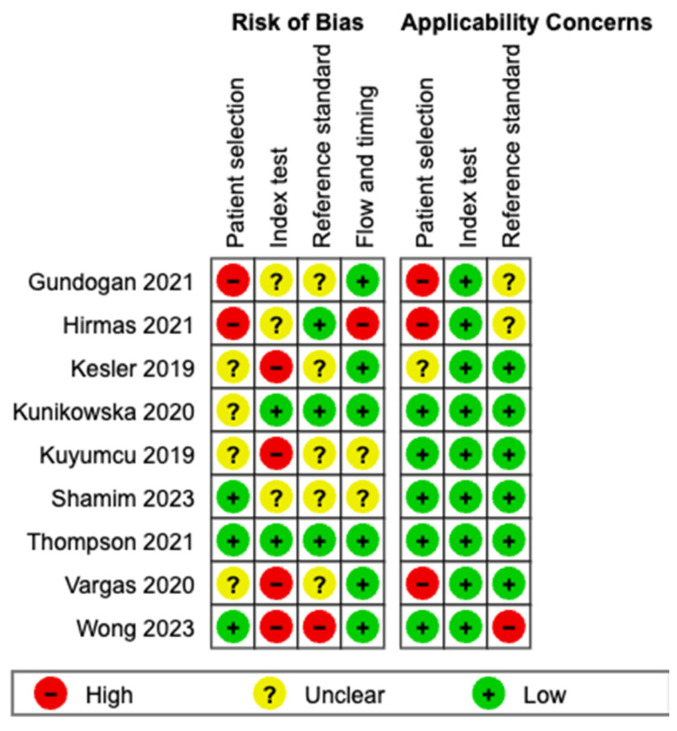
Methodological quality summary per study—QUADAS-2 [21,22,23,24,25,26,27,28,29].

The overall risk of bias was high or unable to be assessed among six of the nine included studies (Figure 2 and Figure 3). Four of these studies [23,24,27,29] lacked thorough description of patient selection criteria. This was due to a lack of description of whether patients were randomly or consecutively enrolled and description of the reasons for exclusion of patients; patient selection after diagnosis was reported in two studies [21,22]. Due to a lack of blinding to prior diagnosis or reference standard results, four studies demonstrated a high risk of bias in the index test domain [24,27,28,29]. In the reference standard domain, six studies had high or unclear risk of bias mainly due to a lack of description as to the method utilised for imaging diagnosis, i.e., LIRADS or guideline-based, or a lack of clarification as to why histological diagnosis was carried out. In one study [28], bias was deemed high due to PSMA PET and diagnostic imaging occurring immediately prior to each other and by the same reviewer. Flow and timing were generally assessed as having a low risk of bias, with the exception of one study [22] that exhibited high risk due to insufficient details regarding patient exclusions and the time interval between the reference standard and the index tests [22].

Applicability scores were generally strong across all studies, with patient selection being the only relevant domain, as three studies expressed high risk of bias [21,22,27]. Within the applicability of the reference standard, Wong et al. [28] was assessed to have a high risk of bias due to diagnostic imaging occurring at time of PSMA PET, with a multi-disciplinary meeting consensus being the standard of truth for HCC diagnosis in the absence of directly recorded LIRADS scores; this and the timing of the imaging introduced bias.

### 3.3. Qualitative Analysis (Systematic Review)

#### 3.3.1. Study and Patient Characteristics

Nine studies were identified during full-text review with sufficient data to analyse PSMA PET in the detection of HCC (Table 1) [21,22,23,24,25,26,27,28,29]. Across these studies, 196 patients with 491 lesions on conventional imaging (CT or MRI) underwent PSMA PET. Studies were published between 2019 and 2023. Countries from the Americas, Middle East, Europe, India, and Australia were represented. Eight of the nine were prospective cohort studies, and one was a retrospective cohort study. Histological diagnosis was made in 70 patients (35.7%), and imaging diagnosis was made in 137 (69.9%). Imaging diagnosis was variably reported: Three studies reported using LIRADS criteria or were guidelines-based [24,26,29]. Wong et al. [28] described LIRADS criteria but used multi-disciplinary team consensus as the standard of truth, while three studies described using imaging for diagnosis but did not clarify the criteria used for this [21,22,25].

#### 3.3.2. PSMA PET Scan Attributes

The characteristics of the included studies’ PET scans can be seen in Table 2. Across the nine included studies, there was relative consistency in type of intervention device, with eight (90%) utilising PET/CT and one study allowing use of both PET/CT and PET/MRI. Radioisotope details were variably reported, although all nine utilised ^68^Ga-PSMA (also reported as ^68^Ga-PSMA-11 or ^68^Ga-PSMA-HBED-CC, all the same tracer). Uptake time varied between 40 to 90 min across studies where it was reported (*n* = 7, 70%). Administered activity was variably reported as median dose or per kg dosing.

#### 3.3.3. Qualitative Results

The qualitative analyses of 196 patients were included (Table 3). Eight studies (*n* = 177 patients) reported per-patient PSMA PET/CT, with 153 (86.4%) demonstrating PSMA uptake in at least one lesion. All studies reported mean SUVmax data, and six studies reported SUVmax data on a per-lesion basis, with 349 lesions reported on conventional imaging and PSMA PET uptake expressed in 292 (83.7%). Five studies reported the same or lower numbers of lesions compared to conventional imaging. One study [23] reported 44 lesions on PSMA PET/CT (152%) compared with 29 on conventional imaging but without histopathology to confirm the discrepancy. Semi-quantitative measures were documented in all studies, with mean SUVmax and tumour-to-background ratio (TBR) reported consistently. The mean SUVmax across studies ranged from 10.8 to 20.9, and the mean TBR ranged from 3.0 to 3.6, indicating an increased SUVmax associated with HCC compared to the background physiological liver uptake.

Diagnosis of HCC was established via reported imaging-based LIRADS or a guideline based on LIRADS or histopathology. Histological confirmation of HCC was performed in 70 patients (39.5%). Imaging-based diagnosis was able to be made in 137 patients (80.1%). The reporting of imaging-based diagnostic methods was heterogenous among the studies; imaging was deemed to be qualitative if the HCC diagnosis was based on consensus opinion without clarification or when including lesions below the standard LIRADS 5 classification of HCC, i.e., inclusion of LIRADS 4 lesions as diagnostic.

**Figure 4:**
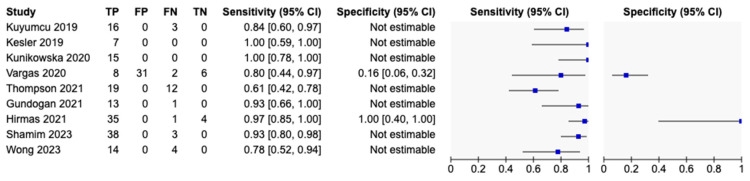
Diagnostic accuracy for PSMA PET for HCC on per-patient analysis [21,22,23,24,25,26,27,28,29]. TP—true positive, FP—false positive, FN—false negative, FP—false positive.

**Figure 5:**
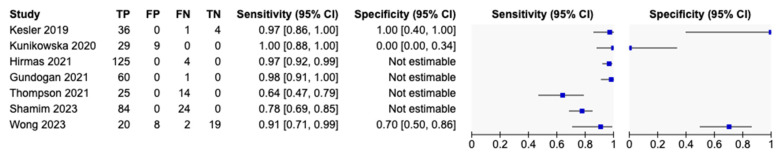
Diagnostic accuracy for PSMA PET for HCC on per-lesion analysis [21,22,23,25,26,28,29]. TP—true positive, FP—false positive, FN—false negative, FP—false positive.

The diagnostic accuracy of the included studies was assessed on a per-patient (Figure 4) and per-lesion (Figure 5) basis. Seven (77.8%) studies reported data on a per-lesion basis, but only three studies reported enough data for specificity to be calculated. There was limited reporting of false-positive results, likely due to positive lesions not being biopsied in the included studies along with a lack of true-negative lesions. The detection rate of HCC (sensitivity) was widely reported; on a per-patient analysis of the nine studies reporting data on sensitivity, this ranged from 61% (95% CI 42–78) to 100% (95% CI 78–100), while specificity was only able to be calculated from two studies and ranged from 16% (95% CI 6.0–32.0) to 100% (95% CI 40.0–100). On the per-lesion analysis, sensitivity was reported with a range from 78% (95% CI 69.0–85.0) to 100% (95% CI 88.0–100). Specificity ranged widely among the three studies, with data reported from 0% (95% CI 0.0–34.0) to 100% (95% CI 40.0–100).

Given the number of patients with a histological diagnosis, it would be ideal to correlate the histologically proven HCC diagnosis with PSMA uptake; however, despite seven of the nine studies including cases with histology, only in two studies [23,27] was it certain which patients these were, and in relation to PSMA uptake data, unfortunately, for the remaining five studies, this was not clarified. Between the two studies, there were 25 cases with histologically proven HCC, and among these, 23 were positive upon PSMA PET, giving a sensitivity of 92%; specificity could not be calculated.

### 3.4. Quantitative Analysis (Meta-Analysis)

Meta-analyses of diagnostic accuracy at a per-lesion and per-patient level were carried out on the nine studies utilising ^68^Ga-PSMA-11 (^68^Ga-PSMA-HBED-CC). The per-patient pooled analysis included 196 patients from the nine studies, while seven studies were included in the per-lesion-based pooled analysis of 491 lesions in 167 patients. The reference standard for diagnosis was histological or LIRADS 5 characteristics.

**Figure 6:**
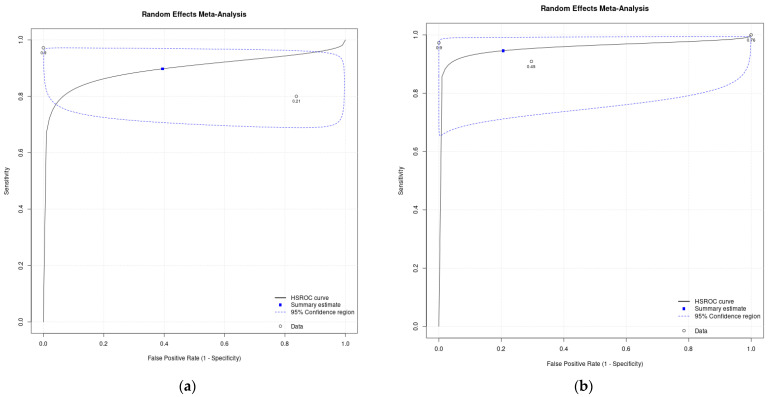
Summary Receiver Operating Curve (SROC): (**a**) per-patient analysis; (**b**) per-lesion analysis.

The per-patient detection rate of PSMA PET for HCC ranged from 61.3% (95% CI 42.2–78.2) to 100% (95% CI–78.2–100), with a pooled estimate of 89.8% (95% CI 78.5–95.5). Per-patient specificity for PSMA for HCC ranged from 16.2% (95% CI 6.2–32.0) to 100% (95% CI 39.8–100), with a pooled estimate of 60.5% (95% CI 1.2–97.2). The HSROC plot of per-patient data demonstrates significant heterogeneity, with a very wide 95% CI for specificity and spread data points (Figure 6a).

The per-lesion detection rate ranged from 64.10% (95% CI 47.2–78.8) to 100% (95% CI 88.1–100), with a pooled estimate of 94.5% (95% CI 82.9–98.4). Per-lesion specificity ranged from 0% (95% CI 0–33.6) to 100% (95% CI 39.8–100), with a pooled estimate of 79.4% (95% CI 2.5–99.8). The HSROC plot of per-lesion data demonstrates significant heterogeneity, with a wide 95% CI region for specificity and spread data points (Figure 6b).

Subgroup analysis was performed on studies with full sensitivity and specificity data. On a per-patient analysis, two studies including 50 patients were analysed, with a pooled estimate for sensitivity of 92.5% (95% CI 64.0–98.9) and pooled estimate for specificity of 72.4% (95% CI 1.3–99.8), LR+ of 3.35 (95% CI 0.1–166.5), LR− of 0.10 (95% CI 0.0–1.9), and DOR of 32.4 (95% 0.0–23,504.3). Per-lesion analysis of the three studies with adequate data represented 115 lesions in 41 patients, with a pooled estimate for sensitivity of 97.1% (95% CI 87.8–99.4) and pooled estimate for specificity of 42.2% (95% CI 0.3–99.4%), LR+ of 1.68 (95% CI 0.2–16.1), LR– of 0.07 (95% CI 0.0–1.1), and DOR of 24.5 (95% CI 0.2–3365.1).

## 4. Discussion

PSMA PET holds significant promise as an imaging modality in HCC. Given the widespread use and availability of PSMA radiotracers, they present an attractive possibility to better image HCC compared with the poorly sensitive ^18^F-FDG PET [5,6,7]. The most common radioisotope used for PSMA PET/CT was ^68^Ga-PSMA-11; the ^68^Ga radioisotope is produced by a ^68^Ge/^68^Ga generator that captures the ^68^Ga as the decay product of germanium-68. This is an ideal production technique when a generator is onsite; however, with a half-life of only 68 min, it is less ideal for transport to distant sites. ^18^F, on the other hand, requires a cyclotron for production, but with a half-life of 110 min, it allows for the production of a longer-lasting radiotracer that is better suited to distant transport and use in peripheral centres. It is exciting to see the study by Michalski et al. as the first study published utilising an ^18^F-tracer (^18^F-PSMA-1007) [20].

Meta-analysis of the available data looking at ^68^Ga-PSMA-11 found that the sensitivity of PSMA PET for HCC is high: 89.8% on the per-patient pooled analysis and 94.5% on the per-lesion pooled analysis. Unlike sensitivity, specificity has been underreported, with pooled analyses of limited relevance given the width of the confidence intervals observed and large-area SROC confidence interval of the summary points. The poor reporting of data within studies to calculate specificity is largely due to the lack of reporting of benign lesions where PSMA uptake was also negative and lack of histology of benign lesions, and few studies have included any data on indeterminate liver lesions. This is important information to help better understand the accuracy of this test. Underreporting of LIRADS observations of individual lesions was also observed, which leads to an inability to compare imaging findings; where a useful tool such as LI-RADS is available, its use assists in producing reproducible reporting. Use of a tool such as LIRADS for qualifying the diagnostic probability of a lesion being HCC should be encouraged to ensure reproducibility and comparability of diagnostic imaging.

Research of PSMA PET for HCC is still in its infancy, and this present meta-analysis established that PSMA PET has an adequate sensitivity to be investigated as a possible candidate for a more reliable PET radiotracer for HCC. Research should now focus on establishing the specificity for HCC in the context of known risk factors. It is likely that PSMA PET will not be specific for HCC compared to other malignancies [13], but it may prove to be specific for malignant over benign tissue. This, taken in context with established risk factors for HCC, i.e., cirrhosis, hepatitis B, and a past history of HCC, may allow a similar probability scale for HCC diagnosis to be developed for PSMA PET as for other contrast-enhanced imaging.

Research into PSMA PET in non-prostatic malignancy is in its early stages, including imaging of HCC. In this context, this systematic review and meta-analysis reports the best data available at this stage; however, it needs to be acknowledged that there are significant limitations to this analysis due to limited studies in this space. Given the low number of clinical trials, we included studies with retrospective data and studies with a high risk of bias, as evident in the quality assessment of these studies. Only one study [26] demonstrated a low risk of bias across all domains assessed. Standardisation of imaging diagnosis should be encouraged in HCC studies to better understand generalisability and applicability of the findings. We attempted to reduce bias in the PSMA PET protocols by excluding one study utilising a unique radio-tracer (^18^F-PSMA-1007) [20], although, even when restricting to the ^68^Ga-PSMA-11 radiotracer, there was inconsistency in the administered dose of the radiotracer, variability in whether this was weight-based or flat, and differences in uptake time. Given the use of a tracer designed for a separate purpose, optimal dose and uptake time were unable to be established based on the data in the included studies. Overall, this study is limited by its ability to confidently report on specificity and thus accurate summary points for the meta-analysis; these have large confidence intervals for this reason, which highlight the need for further rigorous research in this space.

PSMA PET presents an exciting opportunity in HCC, and there have been significant developments in PET technology that are yet to show benefit in HCC. The promise of a PET radiotracer that would be able to accurately identify malignant compared to benign tissue has particular relevance in HCC, given that HCC is a unique cancer that is seldom biopsied. The possibility that PSMA PET may aid in both more accurate staging and possibly monitoring for recurrence of disease in treated patients is the most readily available utility for this technology, especially in a pre-liver transplant population, where biopsy is commonly avoided. However, the most interesting prospect is that of the theranostic potential of PSMA PET. Theranostics, i.e., peptide receptor radionucleotide therapy (PRRT), is a therapeutic strategy whereby the ^68^Ga or ^18^F radioisotope is replaced by the ^177^Lutetium (Lu) isotope, which can deliver beta-particle radiation by binding to the cell, delivering highly targeted radiotherapy to the tumour. This technique was initially developed for treatment of neuroendocrine tumours [30] and has been used more recently in prostate cancer, as demonstrated in the TheraP [11] and VISION [12] trials showing that ^177^Lu-PSMA improved progression-free and overall survival; thus, it is now utilised in the treatment of metastatic castrate-resistant prostate cancer.

Imaging modalities that may delineate tissue biology can assist in diagnosis, prognosis, and very possibly new treatment options in the form of theranostic treatment with ^177^Lu-PSMA. Looking forward, research priorities of high importance based on the analysis are to (1) establish specificity of PSMA PET for HCC in the at-risk population, (2) establish pre- and post-treatment characteristics of PSMA PET uptake, (3) establish PSMA PET uptake within locally advanced and metastatic HCC, (4) investigate the use of ^18^F-based PSMA radioisotopes, and (5) look further at the theranostic development pathway based on these results.

## 5. Conclusions

PSMA PET is a promising imaging modality for HCC that may become useful in ruling out HCC and identifying multifocal or metastatic disease. In this systematic review and meta-analysis, ^68^Ga-PSMA-11demnonstrated a high sensitivity for HCC on a per-patient and per-lesion level. The current literature does not sufficiently report the data needed to accurately assess the specificity of ^68^Ga-PSMA-11 for HCC, and diagnostic accuracy therefore remains unknown.

Further prospective studies are necessary, with a focus on the accurate reporting of benign lesions and inclusion of patients with an intermediate probability of HCC, which will allow accurate reporting of specificity and establish the true diagnostic accuracy of PSMA PET for HCC. PSMA PET holds promise not only as a diagnostic but also possibly a theranostic tool in the management of advanced-stage HCC, which, despite advancements in treatment, has a median overall survival of less than 2 years [31]. Understanding the true diagnostic accuracy of PSMA PET will allow for the design of trials to further assess the accuracy of staging and subsequently use of ^177^Lu-PSMA in HCC.

## Figures and Tables

**Table 1:** Study Baseline Patient Characteristics.

Author	Year	Country	Study Design	No. Patients	No. Lesions	*n* (%) Female	Diagnosis Histological *n* (%)	Diagnosis Imaging (Li-RADS 5) *n* (%)	Mean Age (SD)	Median AFP (Range)	Median Tumour Size mm (Range)	Cirrhosis *n* (%)	HBV *n* (%)
Kuyumcu [24]	2019	Turkey	Prospective	19	NA	3 (15.8)	1 (5.3)	18 (94.7)	57.9 (5.9)	695 (22,050)	NA	10 (52.6)	NA
Kesler [29]	2019	Israel	Prospective	7	37	2 (28.6)	3 (42.8)	7 (100)	55.6 (9.7)	1800 (5328)	NA	4 (57.1)	2 (28.6)
Vargas [27]	2020	Mexico	Prospective	10	NA	NA	10 (100)	NA	NA	NA	NA	NA	NA
Kunikowska [23]	2020	Poland	Prospective	15	29	2 (13.3)	15 (100)	NA	55.6 (18.2)	2756 (69,509)	23 (159)	4 (26.7)	5 (33.3)
Gundogan [21]	2021	Turkey	Prospective	14	57	1 (7.1)	6 (42.8)	8 (57.1)	63.8 (6.0)	148 (60,469)	80.5 (120)	14 (100)	NA
Hirmas [22]	2021	Germany	Retrospective	40	142	6 (15)	20 (50)	20 (50)	NA	36.1 (19,078)	59 (159)	28 (70)	NA
Thompson [26]	2021	USA	Prospective	31	39	8 (25.8)	7 (22.6)	24 (77.4)	NA	4.0 (979.2)	NA	NA	1 (3.2)
Wong [28]	2023	Australia	Prospective	19	49	1 (5)	NA	19 (100)	65 (7.2)	5	19 (45)	19 (100)	0 (0)
Shamim [25]	2023	India	Prospective	41	138	5 (12.2)	8 (19.5)	41 (100)	53.9 (10.9)	243 (46,325)	NA	35 (85)	NA

NA—Not available.

**Table 2:** PSMA PET scan attributes of included studies.

Author	Intervention Device	Radioisotope	Scan Manufacturer	Uptake Time (min) (% Range)	Administered Activity
Kuyumcu [24]	PET/CT	^68^Ga-PSMA	Siemens Healthcare, Biograph TruePoint	60	2.5 (±10%) Mbq/kg
Kesler [29]	PET/CT	^68^Ga-PSMA-11	GE Healthcare Discovery 690	NA	148 Mbq
Vargas [27]	PET/CT	^68^Ga-PSMA	NA	NA	NA
Kunikowska [23]	PET/CT	^68^Ga-PSMA-11	Siemens Biograph 64 TruePoint	60	2.0 Mbq/kg
Gundogan [21]	PET/CT	^68^Ga-PSMA-11	Siemens mCT 20 ultra HD LSO	60	2–2.5 Mbq/kg
Hirmas [22]	PET/CT	^68^Ga-PSMA-11	Siemens Biograph 128mCT; and Siemens Biograph Vision	78 (50–135)	112.5 (79–344) Mbq
Thompson [26]	PET/MRI;PET/CT	^68^Ga-PSMA-11	Siemens Biograph Vision 600; Siemens Healthineers	90 ± 15	NA
Wong [28]	PET/CT	^68^Ga-PSMA-11	GE Discovery	40	60 kg (200 MBq) 61–90 kg (250 MBq) >90 kg (300 MBq)
Shamim [25]	PET/CT	^68^Ga-PSMA	Siemens Biograph mCT 64 PET/CT; GE Discovery 710 PET/CT	50–60	1.8–2.2 Mbq

NA—Not available.

**Table 3:** Qualitative lesion detection rate and PSMA uptake.

Author	No. Patients	No. Lesions (Conventional Imaging)	Histological Diagnosis *n* (%)	Imaging Diagnosis *n* (%)	Positive PSMA Uptake per Patient *n* (%)	Positive PSMA Lesion Uptake *n* (%)	Lesion SUVmax Mean (SD)	TBR Mean (SD)
Kuyumcu [24]	19	NA	1 (5.3)	18 (94.7)	16	NA	17.4 (9.0)	3.3 (2.2)
Kesler [29]	7	37	3 (42.8)	7 (100)	7	36 (97.3)	13.2 (6.7)	3.59 (1.4)
Vargas [27]	10	NA	10 (100)	NA	8 (80)	NA	17.9	NA
Kunikowska [23]	15	29	15 (100)	NA	15 (100)	44 (151.7)	14.7 (6.7)	3.60 (2.1)
Gundogan [21]	14	57	6 (42.8)	8 (57.1)	14 (100)	57 (100)	20.88 (11.8)	3.12 (2.3)
Hirmas [22]	40	142	20 (50)	20 (50)	36 (100)	NA	16.2	NA
Thompson [26]	31	39	7 (22.6)	24 (77.4)	19 (61.3)	26 (66.7)	12.05 (6.9)	2.96 (2.5)
Wong [28]	19	49	NA	19 (100)	NA	28 (57.1)	10.80 (4.9)	NA
Shamim [25]	41	138	8 (19.5)	41 (100)	38 (92.6)	101 (73.2)	11.78 (8.0)	3.17 (2.3)

NA—Not available.

## Data Availability

Data can be made available upon reasonable request to the corresponding author.

## Supplementary Materials

The following supporting information can be downloaded at https://www.mdpi.com/article/10.3390/cancers16223865/s1, Full search strategies.
