# Supplementary material for: Prostate-Specific Membrane Antigen (PSMA) PET/CT in the Detection and Diagnosis of Hepatocellular Carcinoma (HCC): A Systematic Review and Meta-Analysis
Source: Cancers (Basel). 2024 Nov 19;16(22):3865. doi: 10.3390/cancers16223865 (PMC11592426; doi:10.3390/cancers16223865)
Supplement: Supplementary file 1 [file cancers-16-03865-s001.zip › Final search strategies.pdf]

Nicholas Hannah et al. – Systematic Review search

Topic: Prostate Specific Membrane Antigen (PSMA) PET/CT for Diagnosis and Staging of Hepatocellular Carcinoma.

Sources: Medline, EMBASE, Cochrane CENTRAL, CINAHL, Web of Science (November 30, 2023)

Final search strategies

Ovid MEDLINE(R) ALL <1946 to November 28, 2023>

1	carcinoma, hepatocellular/	106700
2	exp liver neoplasms/	196377
3	(Hepatoma* or Hepatoblastoma* or Hepato-blastoma*).mp. [mp=title, book title, abstract, original title, name of substance word, subject heading word, floating sub-heading word, keyword heading word, organism supplementary concept word, protocol supplementary concept word, rare disease supplementary concept word, unique identifier, synonyms, population supplementary concept word, anatomy supplementary concept word]	34977
4	((liver or hepato*) adj3 (cancer* or carcinom* or malign* or metasta* or neoplas* or tumo?r*)).mp.	278209
5	HCC.mp.	78859
6	or/1-5 [LIVER CANCER]	291141
7	prostate-specific membrane antigen*.mp.	4384
8	PSMA.mp.	6003
9	or/7-8 [PSMA]	6657
10	positron emission tomography computed tomography/	20137
11	positron emission tomography-computed tomography.mp.	28266
12	PET-CT.mp.	40680
13	or/10-12 [PET-CT]	47517
14	9 and 13 [PSMA and PET-CT]	2903
15	PSMA PET*.mp.	2023
16	or/14-15 [PSMA PET-CT combined]	3217
17	6 and 16	96

Embase <1974 to 2023 November 28>

1	liver cell carcinoma/	20414<br>7
2	exp liver tumor/	35753<br>5
3	(Hepatoma* or Hepatoblastoma* or Hepato-blastoma*).mp. [mp=title, abstract, heading word, drug trade name, original title, device manufacturer, drug manufacturer, device trade name, keyword heading word, floating subheading word, candidate term word]	44876
4	((liver or hepato*) adj3 (cancer* or carcinom* or malign* or metasta* or neoplas* or tumo?r*)).mp.	41100<br>2
5	HCC.mp.	12297<br>4
6	or/1-5 [LIVER CANCER]	44927<br>0
7	prostate-specific membrane antigen*.mp.	11086
8	PSMA.mp.	11764
9	or/7-8 [PSMA]	14108
10	positron emission tomography-computed tomography/	67100
11	positron emission tomography-computed tomography.mp.	73792
12	PET-CT.mp.	89218
13	or/10-12 [PET-CT]	11391<br>1
14	9 and 13 [PSMA and PET-CT]	6134
15	PSMA PET*.mp.	4525
16	or/14-15 [PSMA PET-CT combined]	7032
17	6 and 16	298

Cochrane CENTRAL – 30/11/2023

ID	Search	Hits
#1	MeSH descriptor: [Carcinoma, Hepatocellular] this term only	2433
#2	MeSH descriptor: [Liver Neoplasms] explode all trees	3968
#3	Hepatoma* or Hepatoblastoma* or Hepato-blastoma*	281
#4	(liver or hepato*) NEAR/3 (cancer* or carcinom* or malign* or metasta* or neoplas* or tumo?r*)	13065
#5	HCC	4316
#6	#1 OR #2 OR #3 OR #4 OR #5	13603
#7	prostate-specific membrane antigen*	251
#8	PSMA	534
#9	#7 OR #8	567
#10	MeSH descriptor: [Positron Emission Tomography Computed Tomography] this term only	308
#11	positron emission tomography-computed tomography	1484
#12	PET-CT	2637
#13	#10 OR #11 OR #12	2910
#14	#9 AND #13	307
#15	PSMA PET*	405
#16	#14 OR #15	415
#17	#6 and #16 in Trials	5

CINAHL – 30/11/2023

#	Query	Results
S1	(MH "Carcinoma, Hepatocellular")	13,815
S2	(MH "Liver Neoplasms+")	23,524
S3	TX hepatoma* OR hepatoblastoma* OR hepato-blastoma*	3,300
S4	TX (liver OR hepato*) N3 (cancer* OR carcinom* OR malign* or metasta* OR neoplas* OR tumo#r*)	49,861
S5	TX HCC	12,507
S6	S1 OR S2 OR S3 OR S4 OR S5	52,124
S7	TX prostate specific membrane antigen* OR PSMA	1,453
S8	(MH "Positron Emission Tomography Computed Tomography")	268
S9	TX positron emission tomography computed tomography OR PET-CT	11,857
S10	S8 OR S9	11,857
S11	S7 AND S10	563
S12	TX PSMA PET*	542
S13	S11 OR S12	805
S14	S6 AND S13	31

Web of Science Core Collection
- WOS.SCI: 1900 to 2023
- WOS.ESCI: 2005 to 2023
- WOS.ISTP: 1990 to 2023

#	Search Query	Results
1	TS=(((prostate-specific membrane antigen* OR PSMA) AND (positron emission tomography-computed tomography OR PET-CT) OR PSMA PET-CT)) Editions: WOS.SCI,WOS.ISTP,WOS.ESCI	4014
2	TS=(((liver or hepato*) NEAR/3 (cancer* or carcinom* or malign* or metasta* or neoplas* or tumo?r*)) OR HCC OR hepatoma* OR hepatoblastoma*) Editions: WOS.SCI,WOS.ISTP,WOS.ESCI	294677
3	#2 AND #1 Editions: WOS.SCI,WOS.ISTP,WOS.ESCI	71
